# High-Performance Asymmetric Supercapacitors Assembled from La-Doped ZnCo_2_O_4_/MnCo-LDH Nanoflower Positive Electrodes and Ti-Supported Sb-Doped SnO_2_ Negative Electrodes

**DOI:** 10.3390/mi17060692

**Published:** 2026-06-03

**Authors:** Wei Xu, Changxu Qu, Mingzhao Xing, Jing Wang, Yanzhi Sun

**Affiliations:** School of Light Industry, Harbin University of Commerce, Harbin 150028, China

**Keywords:** La-doped ZnCo_2_O_4_/MnCo-LDH, Ti/Sb-SnO_2_, defect regulation, asymmetric supercapacitors

## Abstract

Transition-metal oxide/layered double hydroxide (LDH) electrodes often suffer from insufficient utilization of active sites, sluggish electron/ion transport, and limited cycling stability at high rates. Here, La-doped ZnCo_2_O_4_/MnCo-LDH nanoflowers serve as the positive electrode and Ti-supported Sb-doped SnO_2_ (Ti/Sb-SnO_2_) serves as the negative electrode for constructing an asymmetric supercapacitor. A stepwise hydrothermal route, La-doping regulation, and ethylenediamine-assisted morphology control transform stacked nanosheets into open porous nanoflowers with a specific surface area of 382.5 m^2^ g^−1^, thereby exposing more electroactive sites and shortening OH^−^ diffusion pathways. La^3+^-induced lattice distortion and defect-related oxygen species further tune the electronic structure and improve interfacial charge-transfer kinetics. The optimized La-ZnCo_2_O_4_/MnCo-LDH electrode delivers 2130 F g^−1^ at 1 A g^−1^ and retains 1993 F g^−1^ after 10,000 cycles at 3 A g^−1^. The Ti/Sb-SnO_2_ negative electrode provides 673 F g^−1^ at 1 A g^−1^ and 302 F g^−1^ at 15 A g^−1^. The assembled device operates stably from 0 to 1.8 V in 2 M KOH and achieves 69 Wh kg^−1^ and 13,500 W kg^−1^.

## 1. Introduction

With the rapid development of renewable-energy integration, portable electronics, and electric transportation, electrochemical energy-storage devices that combine high power density, long cycle life, and competitive energy density have attracted sustained attention [1,2,3,4,5,6,7]. Supercapacitors offer fast charge–discharge behavior and high-power output; however, traditional electrical double-layer capacitors are limited by low energy density, while single pseudocapacitive materials are frequently constrained by poor conductivity, slow ion diffusion, and structural degradation during repeated cycling. Therefore, the synergistic design of multicomponent composites, defect regulation, and three-dimensional open architectures is an effective strategy for improving electrode reaction kinetics and device-level performance.

Transition-metal oxides and layered double hydroxides (LDHs) are widely investigated as pseudocapacitive electrodes because of their tunable valence states, abundant redox activity, and high theoretical capacitance. ZnCo_2_O_4_ provides favorable structural stability and multivalent redox activity, whereas MnCo-LDH supplies layered ion-transport channels and rich surface-active sites. Their integration is expected to build a coupled electron/ion transport network [8,9,10,11,12,13]. Nevertheless, nanosheet-based materials tend to restack, which reduces the effective surface area and the number of accessible active sites. Rare-earth La, with a distinctive 4f electronic configuration, can induce lattice distortion, tune electronic structure, and promote the formation of defect-related oxygen species when introduced at an appropriate level, thus improving charge transfer and redox activity [14,15,16,17]. In addition, ethylenediamine can regulate crystal growth and induce the evolution of nanosheets into a more open flower-like porous architecture, which enhances electrolyte wetting and ion-diffusion efficiency.

For asymmetric supercapacitors, the potential window, capacity, and cycling stability of the negative electrode directly affect the output voltage and energy density [18,19,20,21,22,23,24]. SnO_2_ is attractive because of its abundance, chemical stability, and high theoretical capacity, but its intrinsic conductivity and reaction kinetics require improvement. Sb doping can enhance electron transport by lattice substitution, carrier-concentration modulation, and defect regulation, whereas a Ti sheet can function as both a conductive support and a current collector to improve interfacial contact and mechanical robustness [25,26,27,28,29,30]. Based on the available characterization results, the negative electrode is described in this work as Ti-supported Sb-doped SnO_2_ (Ti/Sb-SnO_2_); the Ti signal is not directly assigned to Ti incorporation into the SnO_2_ lattice.

Although ZnCo_2_O_4_/LDH composites have been widely explored as promising pseudocapacitive electrodes, their practical electrochemical performance is still restricted by nanosheet aggregation, insufficient exposure of active sites, limited electronic conductivity, and sluggish ion diffusion during high-rate charge–discharge processes. Therefore, developing a rationally engineered ZnCo_2_O_4_/LDH-based heterostructure with simultaneously optimized electronic structure, defect chemistry, and open ion-transport pathways is highly important for achieving high-performance asymmetric supercapacitors. In this regard, the La-ZnCo_2_O_4_/MnCo-LDH system proposed in this work provides a distinctive design strategy. The ZnCo_2_O_4_/MnCo-LDH heterointerface can couple multivalent redox activity with layered ion-transport channels, while La^3+^ introduction induces local lattice distortion and defect-related oxygen species to regulate the electronic structure and enhance charge-transfer kinetics. Meanwhile, ethylenediamine-assisted morphology control promotes the transformation of stacked nanosheets into an open porous nanoflower architecture, which exposes more electroactive sites and shortens OH^−^ diffusion pathways. Thus, the importance and novelty of this system arise from the synergistic integration of heterostructure engineering, rare-earth-induced defect regulation, and three-dimensional porous morphology design.

Herein, La-ZnCo_2_O_4_/MnCo-LDH nanoflowers and Ti/Sb-SnO_2_ were prepared as positive and negative electrodes, respectively. Their morphology, crystal structure, surface chemical state, pore structure, and electrochemical performance were systematically investigated. A La-ZnCo_2_O_4_/MnCo-LDH//Ti/Sb-SnO_2_ asymmetric supercapacitor was further assembled to clarify the synergistic effects of heterostructure construction, La-induced defect regulation, ethylenediamine-assisted nanoflower morphology, and positive/negative electrode charge matching on energy-storage performance [31,32,33,34,35,36,37].

## 2. Materials and Methods

### 2.1. Preparation of La-ZnCo_2_O_4_/MnCo-LDH Positive Electrode Materials

**Figure 1:**
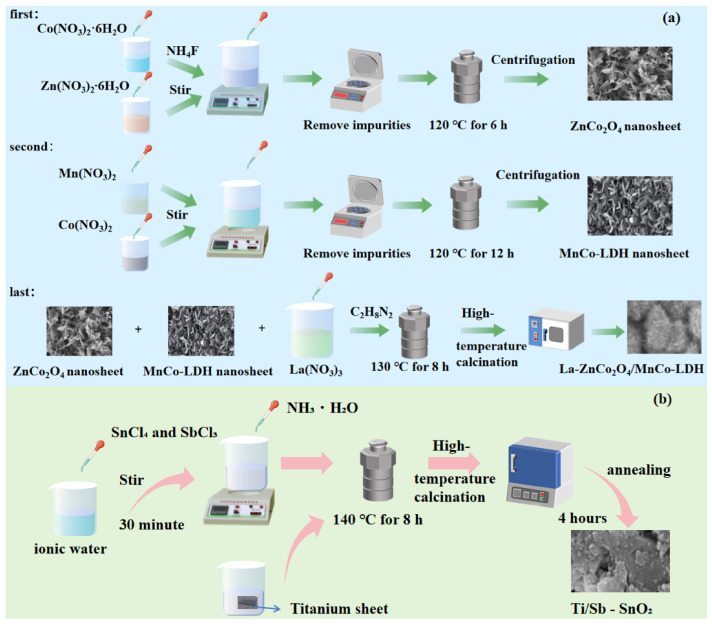
Schematic illustration of the preparation processes for (**a**) the La-ZnCo_2_O_4_/MnCo-LDH nanoflower composite and (**b**) the Ti-supported Sb-doped SnO_2_ (Ti/Sb-SnO_2_) negative electrode.

La-ZnCo_2_O_4_/MnCo-LDH composites were synthesized through a stepwise hydrothermal process combined with doping regulation, as illustrated in Figure 1a. ZnCo_2_O_4_ nanosheets were first prepared by dissolving 1.5 mmol Co(NO_3_)_2_·6H_2_O, 0.75 mmol Zn(NO_3_)_2_·6H_2_O, 7.5 mmol urea, and 4.5 mmol NH_4_F in 60 mL deionized water. After stirring to obtain a homogeneous solution, the mixture was transferred into a Teflon-lined autoclave and heated at 120 °C for 6 h. The product was washed with deionized water and absolute ethanol and then annealed at 350 °C for 2 h to obtain ZnCo_2_O_4_ nanosheets with an active-material loading of approximately 1 mg cm^−2^.

MnCo-LDH nanosheets were prepared through a similar hydrothermal route. Briefly, 1.0 mmol Mn(NO_3_)_2_·4H_2_O, 2.0 mmol Co(NO_3_)_2_·6H_2_O, 6.0 mmol urea, and 3.0 mmol NH_4_F were dissolved in 60 mL deionized water and stirred for 30 min. The solution was then sealed in an autoclave and maintained at 120 °C for 12 h. After cooling, the product was alternately washed with deionized water and absolute ethanol, dried under vacuum at 60 °C for 12 h, and annealed at 200 °C for 3 h under Ar to obtain MnCo-LDH nanosheets with a loading of approximately 1.2 mg cm^−2^.

For the La-doped composite, La(NO_3_)_3_ solution was added to the ZnCo_2_O_4_ and MnCo-LDH precursor dispersion, followed by the introduction of ethylenediamine (C_2_H_8_N_2_) as a morphology-directing agent. The mixture was stirred for 3 h and then transferred to an autoclave for reaction at 130 °C for 8 h. The resulting product was collected by centrifugation, alternately washed with deionized water and ethanol, and dried under vacuum at 80 °C to obtain porous La-ZnCo_2_O_4_/MnCo-LDH nanoflower composites.

### 2.2. Preparation of Ti/Sb-SnO_2_ Negative Electrode Materials

Ti/Sb-SnO_2_ negative electrodes were prepared by a hydrothermal-annealing method, as shown in Figure 1b. SnCl_4_ and SbCl_3_ were dissolved in 60 mL deionized water, with the amount of SnCl_4_ set to 0.1 mol and the Sb/Sn molar ratio set to 0.05. After stirring for 30 min, NH_3_·H_2_O was added dropwise to adjust the pH to approximately 8–9 and promote metal-ion hydrolysis. The pretreated Ti sheet was polished with abrasive paper, ultrasonically cleaned in acetone, immersed in dilute hydrochloric acid to remove the surface oxide layer, and then completely immersed in the precursor solution. The system was transferred into an autoclave and maintained at 140 °C for 8 h to grow the Sb-doped SnO_2_ precursor in situ on the Ti sheet. After the reaction, the Ti sheet was washed with deionized water and absolute ethanol and finally annealed in air at 550 °C for 4 h to obtain the Ti-supported Sb-doped SnO_2_ negative electrode.

### 2.3. Device Assembly and Electrochemical Calculations

The asymmetric supercapacitor was assembled using La-ZnCo_2_O_4_/MnCo-LDH as the positive electrode, Ti/Sb-SnO_2_ as the negative electrode, and 2 M KOH as the electrolyte. To ensure charge balance between the two electrodes, the active-material masses were matched according to q^+^ = q^−^. The specific capacitance, stored charge, mass ratio, energy density, and power density were calculated using Equations (1)–(5) according to previously reported methods [38]:

C_s_ = IΔt/(mΔV) (1)

q = C_s_ × m × ΔV (2)

m^+^/m^−^ = (C_s_^−^ × ΔV^−^)/(C_s_^+^ × ΔV^+^) (3)

E = C_device(ΔV)^2^/7.2 (4)

P = 3600E/Δt(5)

where C_s_ (F g^−1^) is the specific capacitance, I (A) is the discharge current [39], Δt (s) is the discharge time, m (g) is the mass of active material, ΔV (V) is the potential window after subtracting the IR drop, q (C) is the stored charge, and E (Wh kg^−1^) and P (W kg^−1^) are the energy and power densities, respectively. In the three-electrode calculations, m refers to the active-material mass of a single electrode. For the two-electrode device, C_device was calculated based on the total active mass of the positive and negative electrodes.

### 2.4. Characterization and Electrochemical Measurements

The morphology and microstructure of the materials were characterized by scanning electron microscopy (SEM; SU8010, Hitachi High-Technologies Corp., Tokyo, Japan), transmission electron microscopy (TEM; JEM-2100F, JEOL Ltd., Tokyo, Japan), and high-resolution TEM (HRTEM; JEM-2100F, JEOL Ltd., Tokyo, Japan). Elemental composition and distribution were analyzed by energy-dispersive X-ray spectroscopy (EDS; X-Max 80, Oxford Instruments, Abingdon, UK). Crystal structure, pore structure, and surface chemical states were examined by X-ray diffraction (XRD; D8 Advance, Bruker AXS GmbH, Karlsruhe, Germany), N2 adsorption–desorption analysis (ASAP 2460, Micromeritics Instrument Corp., Norcross, GA, USA), and X-ray photoelectron spectroscopy (XPS; ESCALAB 250Xi, Thermo Fisher Scientific, Waltham, MA, USA), respectively. XRD patterns were analyzed using Jade software (version 6.5, Materials Data, Inc., Livermore, CA, USA). XPS spectra were calibrated with reference to the C 1s peak at 284.8 eV and fitted using XPSPEAK software (version 4.1). Three-electrode measurements were conducted using Hg/HgO as the reference electrode, a Pt plate as the counter electrode, and the prepared electrode materials as the working electrodes. Electrochemical performance was evaluated by cyclic voltammetry (CV), galvanostatic charge–discharge (GCD), and electrochemical impedance spectroscopy (EIS) using a CHI 660E electrochemical workstation (Shanghai Chenhua Instrument Co., Ltd., Shanghai, China) [40,41]. EIS spectra were fitted using ZView software (version 3.5, Scribner Associates Inc., Southern Pines, NC, USA), and all electrochemical data and figures were processed using OriginPro 2021 software (version 9.8, OriginLab Corp., Northampton, MA, USA).

## 3. Results and Discussion

### 3.1. Morphology and Structure of the La-ZnCo_2_O_4_/MnCo-LDH Positive Electrode

**Figure 2:**
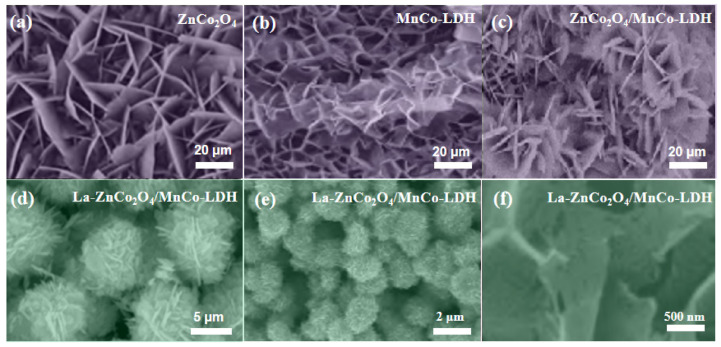
SEM images of (**a**) ZnCo_2_O_4_ nanosheets, (**b**) MnCo-LDH nanosheets, (**c**) ZnCo_2_O_4_/MnCo-LDH composite nanosheets, and (**d**–**f**) La-ZnCo_2_O_4_/MnCo-LDH nanoflower materials.

Figure 2 presents the morphology evolution of ZnCo_2_O_4_, MnCo-LDH, ZnCo_2_O_4_/MnCo-LDH, and La-ZnCo_2_O_4_/MnCo-LDH. Both ZnCo_2_O_4_ and MnCo-LDH exhibit nanosheet structures, and the composite shows interlaced nanosheets. After the introduction of La(NO_3_)_3_ and ethylenediamine, the sheet-like precursors gradually transform into a flower-like porous architecture assembled from ultrathin nanosheets. This structure greatly increases the electrode–electrolyte contact area and provides more accessible active sites for OH^−^ diffusion and pseudocapacitive reactions [42].

**Figure 3:**
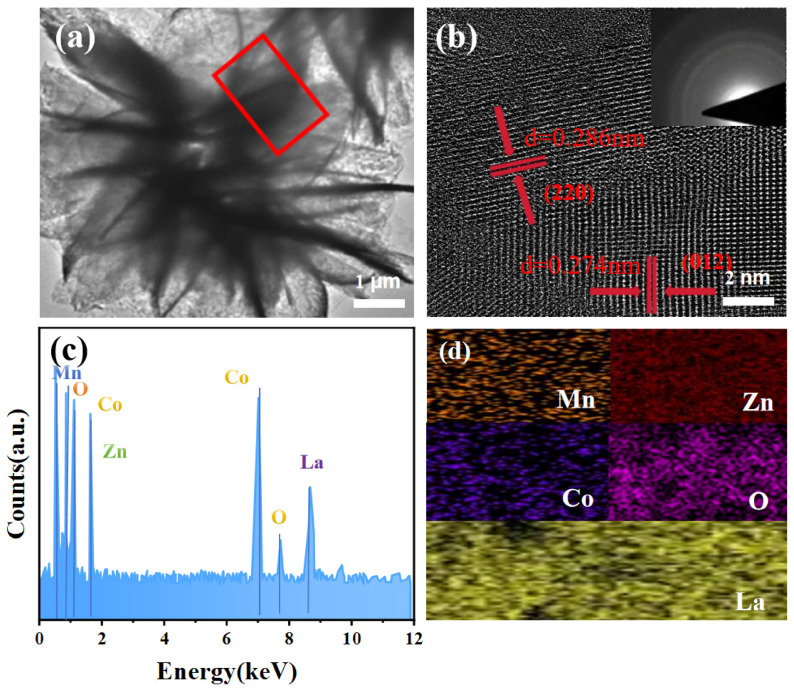
(**a**,**b**) TEM/HRTEM images, (**c**) EDS spectrum, and (**d**) elemental mapping images of Zn, Co, Mn, La, and O for the La-ZnCo_2_O_4_/MnCo-LDH nanoflower composite.

TEM and HRTEM observations further confirm the open nanoflower structure of La-ZnCo_2_O_4_/MnCo-LDH (Figure 3). The low-magnification TEM image reveals a petal-like framework assembled from thin nanosheets. Lattice spacings of 0.286 and 0.274 nm observed in the HRTEM image can be assigned to ZnCo_2_O_4_-related planes and planes in the LDH/oxide composite system, respectively. The selected-area electron diffraction pattern indicates a polycrystalline feature. EDS and elemental mapping results show homogeneous distributions of Zn, Co, Mn, La, and O, confirming that La was successfully introduced into the ZnCo_2_O_4_/MnCo-LDH composite. The uniform La distribution indicates homogeneous La introduction in the nanoflower architecture rather than macroscopic La-rich aggregation; however, EDS mapping alone is not used as sole evidence for substitutional doping.

**Figure 4:**
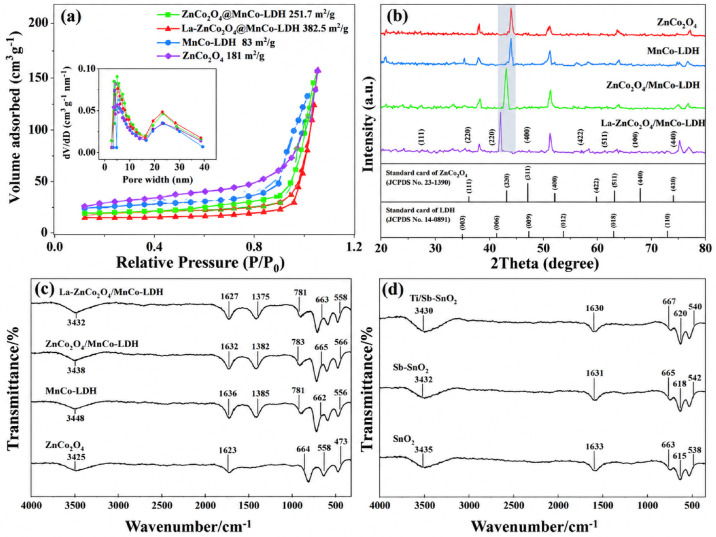
(**a**) N_2_ adsorption–desorption isotherms and pore-size distributions, (**b**) XRD patterns, and FTIR spectra of (**c**) ZnCo_2_O_4_, MnCo-LDH, ZnCo_2_O_4_/MnCo-LDH, and La-ZnCo_2_O_4_/MnCo-LDH positive-electrode materials and (**d**) SnO_2_, Sb-SnO_2_, and Ti/Sb-SnO_2_ negative-electrode materials.

The pore and crystal structures are summarized in Figure 4. The N_2_ adsorption–desorption isotherms of all samples show typical mesoporous characteristics. The specific surface area of La-ZnCo_2_O_4_/MnCo-LDH reaches 382.5 m^2^ g^−1^, which is considerably higher than those of ZnCo_2_O_4_/MnCo-LDH (251.7 m^2^ g^−1^), ZnCo_2_O_4_ (181 m^2^ g^−1^), and MnCo-LDH (83 m^2^ g^−1^). The enlarged surface area and mesoporous channels facilitate electrolyte wetting, shorten ion-diffusion distances, and improve utilization of electroactive sites. XRD patterns show no obvious impurity peaks, and the ZnCo_2_O_4_-related peaks match JCPDS No. 23-1390, while the LDH-related features are consistent with JCPDS No. 14-0191. After compositing and La introduction, some diffraction peaks slightly shift, suggesting that heterocomponent coupling and La introduction may induce local lattice distortion.

FTIR spectra were further collected to support the structural analysis of the synthesized materials. As shown in Figure 4c, the broad absorption bands at approximately 3425–3448 cm^−1^ are attributed to the stretching vibration of O–H groups from surface hydroxyls and adsorbed/interlayer water, while the bands at approximately 1623–1636 cm^−1^ correspond to the bending vibration of H–O–H. The absorption bands around 1375–1385 cm^−1^ can be related to residual interlayer anions or surface carbonate/nitrate species. In the low-wavenumber region, the bands at approximately 781–783, 662–665, 556–558, and 473 cm^−1^ are assigned to metal–oxygen related vibrations, indicating the presence of Zn–O, Co–O, and Mn–O bonding environments in the oxide/LDH composite. After La introduction, these characteristic bands are retained with slight shifts, suggesting that La doping and heterostructure construction do not destroy the main ZnCo_2_O_4_/MnCo-LDH framework but may modify the local bonding environment.

For the negative-electrode materials, the FTIR spectra in Figure 4d show broad O–H stretching bands at approximately 3430–3435 cm^−1^ and H–O–H bending bands at approximately 1630–1633 cm^−1^. The absorption bands located at approximately 663–667, 615–620, and 538–542 cm^−1^ are associated with Sn–O/Sb–O related lattice vibrations. The similar characteristic bands observed for SnO_2_, Sb-SnO_2_, and Ti/Sb-SnO_2_ indicate that the SnO_2_-based oxide framework is maintained after Sb doping and Ti-substrate-supported growth. These FTIR results further support the successful synthesis of La-ZnCo_2_O_4_/MnCo-LDH and Ti/Sb-SnO_2_, and they are consistent with the XRD and XPS results.

**Figure 5:**
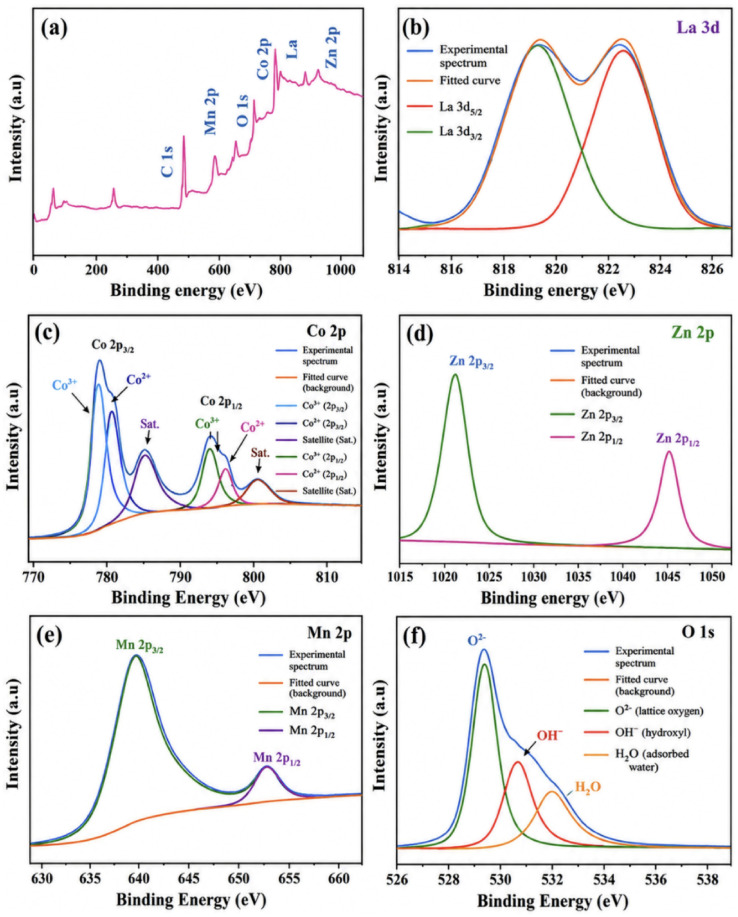
(**a**) XPS survey spectrum and (**b**–**f**) high-resolution La 3d, Co 2p, Zn 2p, Mn 2p, and O 1s XPS spectra of La-ZnCo_2_O_4_/MnCo-LDH.

XPS analysis was conducted to clarify the surface chemical states of La-ZnCo_2_O_4_/MnCo-LDH (Figure 5). The survey spectrum in Figure 5a shows clear La, Zn, Co, Mn, O, and C signals, confirming the coexistence of the main constituent elements in the La-ZnCo_2_O_4_/MnCo-LDH composite. The C signal is mainly attributed to surface adventitious carbon used for charge correction. The high-resolution La 3d spectrum in Figure 5b indicates the presence of La-related bonding environments, suggesting that La is successfully introduced into the composite and participates in local La–O coordination.

As shown in Figure 5c, the Co 2p spectrum reveals the coexistence of Co^2+^ and Co^3+^ species, accompanied by satellite peaks, indicating the mixed-valence nature of cobalt in the composite. The Co^2+^/Co^3+^ redox couple is beneficial for reversible Faradaic reactions and contributes to the enhanced pseudocapacitive behavior. The Zn 2p_3_/_2_ and Zn 2p_1_/_2_ peaks located at approximately 1021.8 and 1044.9 eV are consistent with the characteristic signals of Zn^2+^, confirming the presence of ZnCo_2_O_4_-related components. The Mn 2p spectrum further verifies the existence of Mn-containing LDH/oxide species, which can provide additional redox-active sites and layered ion-transport channels.

The O 1s spectrum can be deconvoluted into lattice oxygen, defect/hydroxyl oxygen, and adsorbed water components. Among them, lattice oxygen corresponds to metal–oxygen bonds in the oxide/LDH framework, while the defect/hydroxyl oxygen component is closely related to oxygen vacancies, surface hydroxyl groups, and defect-related oxygen species. The presence of these oxygen-related species indicates that La introduction and heterostructure construction can regulate the local electronic structure and generate more electrochemically active sites. These defect/hydroxyl oxygen species are favorable for improving electrolyte wettability, promoting OH^−^ adsorption/diffusion, and accelerating interfacial charge-transfer kinetics. Therefore, the detailed XPS analysis further supports that La-induced defect regulation plays an important role in enhancing the charge-storage performance of the La-ZnCo_2_O_4_/MnCo-LDH electrode.

### 3.2. Electrochemical Performance of the La-ZnCo_2_O_4_/MnCo-LDH Positive Electrode

**Figure 6:**
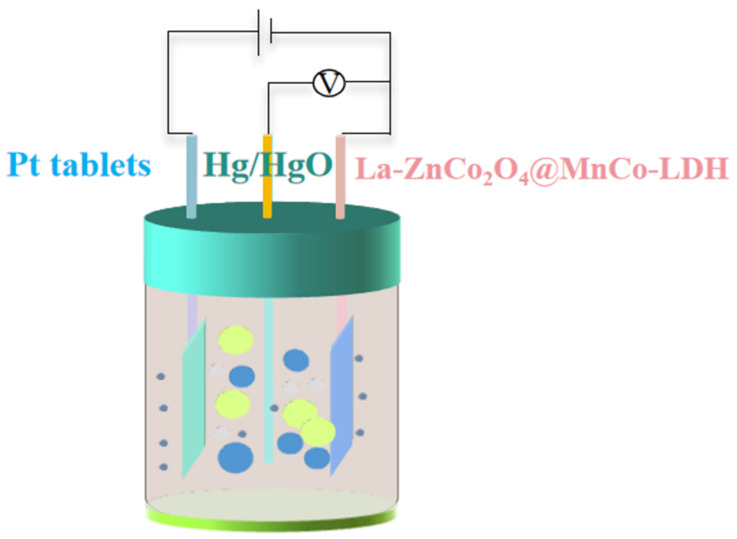
Schematic diagram of the three-electrode measurement system.

**Figure 7:**
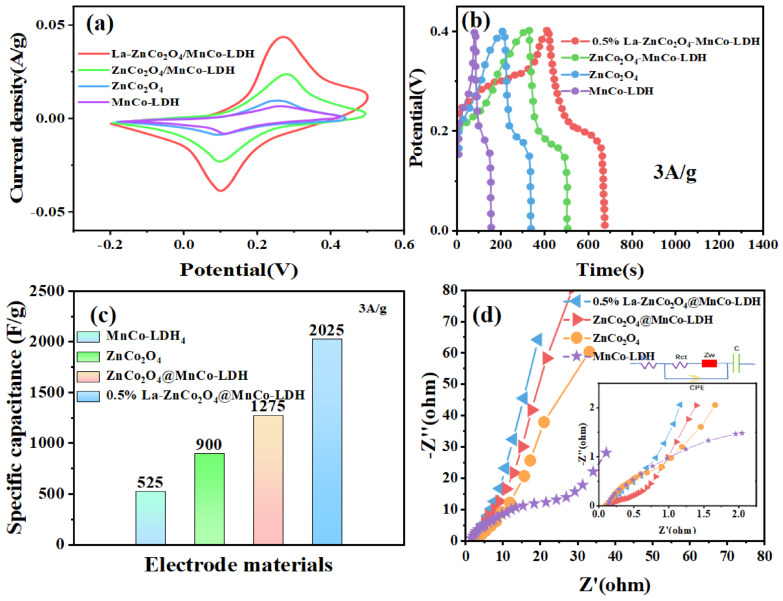
(**a**) CV curves, (**b**) GCD curves, (**c**) specific-capacitance comparison, and (**d**) EIS spectra of La-ZnCo_2_O_4_/MnCo-LDH and comparison samples.

The three-electrode testing configuration is shown in Figure 6. Compared with ZnCo_2_O_4_, MnCo-LDH, and the undoped composite, La-ZnCo_2_O_4_/MnCo-LDH exhibits the largest CV-enclosed area and the longest GCD discharge time (Figure 7), indicating markedly enhanced charge-storage capability. At 3 A g^−1^, the 0.5% La-ZnCo_2_O_4_/MnCo-LDH electrode delivers a specific capacitance of 2025 F g^−1^, far exceeding MnCo-LDH (525 F g^−1^), ZnCo_2_O_4_ (900 F g^−1^), and ZnCo_2_O_4_/MnCo-LDH (1275 F g^−1^). The smaller semicircle diameter and more vertical low-frequency line in the EIS spectra demonstrate lower charge-transfer resistance and faster ion-diffusion kinetics for the La-doped composite electrode. The Nyquist plots were fitted using the equivalent circuit shown in the inset of Figure 7d. The circuit consists of Rs, Rct, CPE, and Zw, where Rs represents the solution/internal resistance, Rct corresponds to the charge-transfer resistance at the electrode/electrolyte interface, CPE describes the non-ideal capacitive behavior caused by the porous and rough electrode surface, and Zw represents the Warburg diffusion impedance associated with electrolyte ion diffusion. This equivalent circuit is suitable for the present EIS spectra because the high-frequency intercept, semicircle region, and low-frequency inclined line correspond to internal resistance, interfacial charge-transfer behavior, and ion-diffusion processes, respectively. The good agreement between the fitted curves and the experimental Nyquist plots confirms the validity of the selected equivalent circuit.

**Figure 8:**
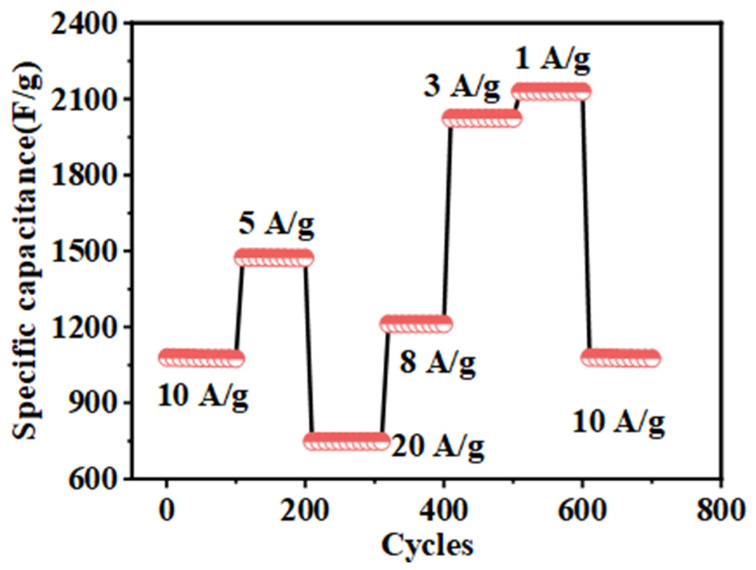
Rate performance of the La-ZnCo_2_O_4_/MnCo-LDH electrode at different current densities.

Rate-cycling results (Figure 8) show that La-ZnCo_2_O_4_/MnCo-LDH maintains good capacitance recovery under continuously varied current densities. When the current density returns to 10 A g^−1^, the specific capacitance recovers from approximately 1080 to 1063 F g^−1^, confirming that the porous nanoflower architecture preserves a stable electron/ion transport network after high-current operation.

**Figure 9:**
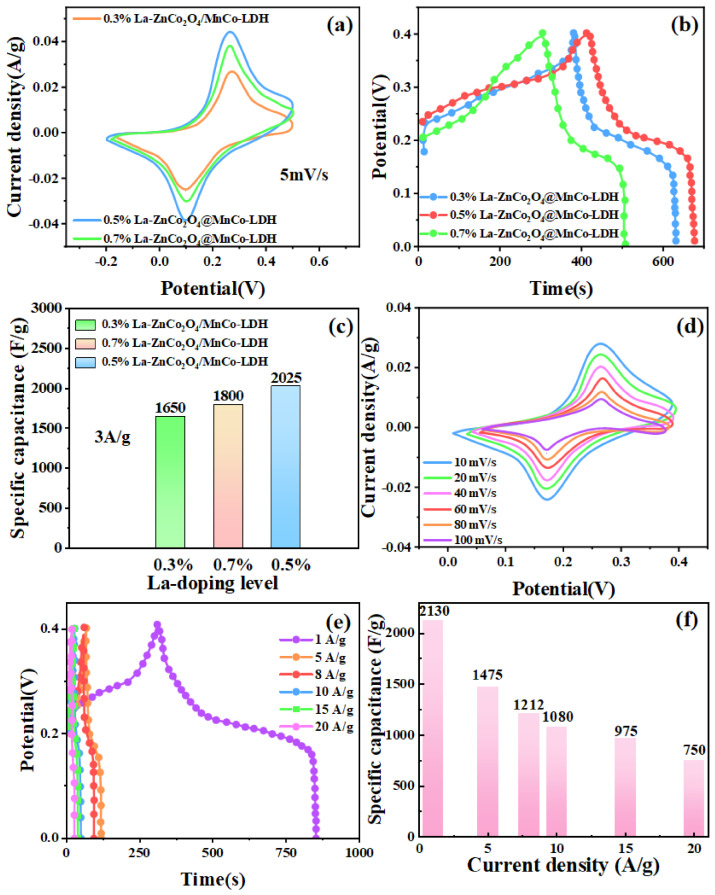
Electrochemical performance of La–ZnCo_2_O_4_/MnCo-LDH electrodes with different La-doping levels: (**a**) CV curves at a scan rate of 5 mV s^−1^; (**b**) GCD curves at a current density of 3 A g^−1^; (**c**) specific capacitances of electrodes with different La-doping levels at 3 A g^−1^; (**d**) CV curves of the optimized 0.5% La–ZnCo_2_O_4_/MnCo-LDH electrode at different scan rates; (**e**) GCD curves of the optimized 0.5% La–ZnCo_2_O_4_/MnCo-LDH electrode at different current densities; and (**f**) rate performance of the optimized 0.5% La–ZnCo_2_O_4_/MnCo-LDH electrode.

To optimize the La-introduction level, the electrochemical behaviors of 0.3%, 0.5%, and 0.7% La-ZnCo_2_O_4_/MnCo-LDH samples were compared (Figure 9). These three nominal La-introduction levels were selected as low, moderate, and relatively high precursor-addition levels to screen the influence of La-related defect regulation on morphology evolution and electrochemical performance. Because quantitative ICP analysis was not performed and trace La quantification by EDS is semi-quantitative, these percentages are reported as nominal La-introduction levels rather than exact measured atomic concentrations; EDS mapping and XPS are used to confirm homogeneous La introduction and La-related bonding. The 0.5% La-doped sample shows higher peak current, longer discharge time, and higher specific capacitance. It delivers 2130 F g^−1^ at 1 A g^−1^, retains 2025 F g^−1^ at 3 A g^−1^, and maintains 750 F g^−1^ even at 20 A g^−1^. These results indicate that an appropriate La-introduction content optimizes defect concentration and charge-transport channels, while excessive introduction may hinder structural and interfacial transport.

**Figure 10:**
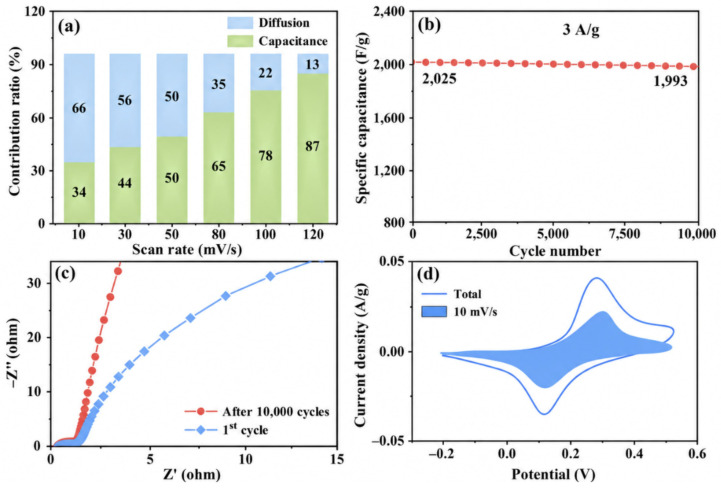
Electrochemical performance and charge-storage analysis of the La-ZnCo_2_O_4_/MnCo-LDH electrode: (**a**) capacitive and diffusion-controlled contribution ratios at different scan rates; (**b**) cycling stability at 3 A/g for10,000 cycles; (**c**) EIS comparison before and affer 10,000 cycles; and (**d**) capacitive contribution analysis at 10 mV/s.

Charge-storage kinetics and cycling stability were further evaluated, as shown in Figure 10. To further distinguish the capacitive-controlled and diffusion-controlled charge-storage behaviors, the b-value was calculated according to the relationship i = av^b^, where i represents the peak current, v is the scan rate, a is an adjustable parameter, and b reflects the charge-storage kinetics. By taking the logarithm of both sides, the equation can be expressed as log(i) = b log(v) + log(a), and the b-value was obtained from the slope of the log(i) versus log(v) plot. Generally, b = 0.5 indicates a diffusion-controlled process, whereas b = 1.0 corresponds to a capacitive-controlled process. The calculated b-values for the anodic and cathodic peaks are 0.48 and 0.49, respectively, suggesting that the electrochemical reaction of the La-ZnCo_2_O_4_/MnCo-LDH electrode is mainly governed by diffusion-controlled Faradaic processes with partial capacitive-controlled contributions. As the scan rate increases from 10 to 120 mV s^−1^, the diffusion-controlled contribution decreases, whereas the capacitive contribution increases from 34% to 87%, indicating that fast surface/near-surface reactions dominate at high scan rates. The cycling test shows that the specific capacitance decreases only from 2025 to 1993 F g^−1^ after 10,000 cycles at 3 A g^−1^. The small change in the EIS spectra before and after cycling further verifies the robust structure and stable interfacial transport behavior of La-ZnCo_2_O_4_/MnCo-LDH.

**Figure 11:**
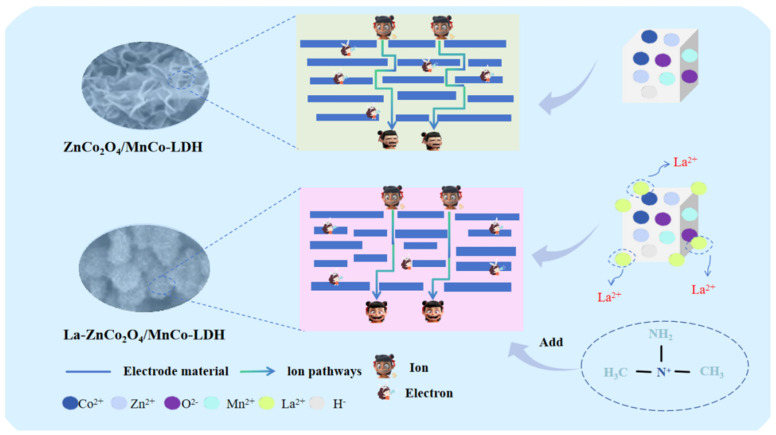
Schematic diagram of the structural evolution and energy-storage mechanism of ZnCo_2_O_4_/MnCo-LDH and La-ZnCo_2_O_4_/MnCo-LDH.

Based on the morphology, structure, and electrochemical results, the performance enhancement of La-ZnCo_2_O_4_/MnCo-LDH can be attributed to the following synergistic effects (Figure 11): (i) the ZnCo_2_O_4_/MnCo-LDH heterostructure balances electron transport and ion diffusion; (ii) La^3+^ introduction induces local lattice distortion and defect-related oxygen species, increasing active sites and improving the electronic structure; (iii) ethylenediamine promotes the transformation of nanosheets into flower-like porous structures, significantly improving surface area and electrolyte accessibility; and (iv) the open pores shorten OH^−^ transport pathways and promote fast, reversible Faradaic reactions.

### 3.3. Structure and Electrochemical Performance of the Ti/Sb-SnO_2_ Negative Electrode

**Figure 12:**
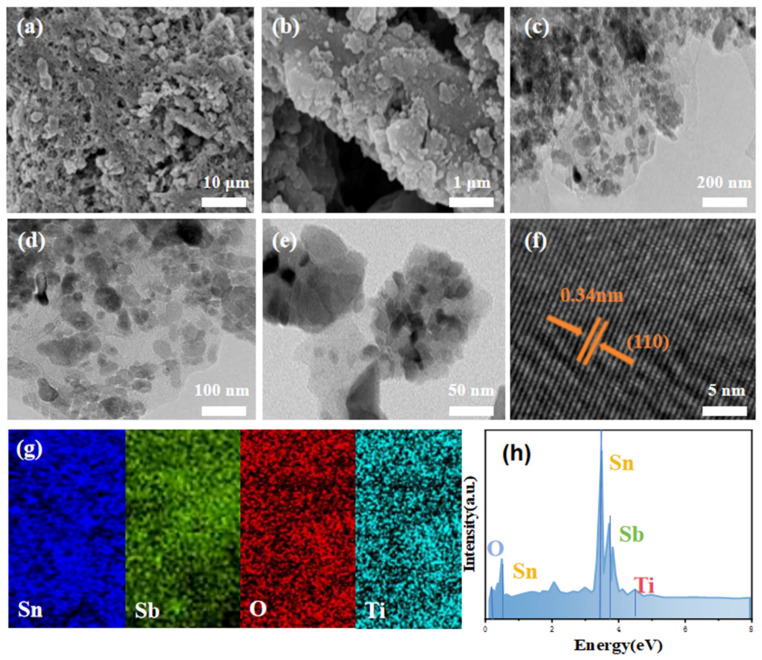
(**a**,**b**) SEM images, (**c**–**e**) TEM images, (**f**) HRTEM image, and (**g**,**h**) elemental mapping and EDS spectrum of Ti/Sb-SnO_2_.

The microstructure of the Ti/Sb-SnO_2_ negative electrode is shown in Figure 12. SEM and TEM images indicate that the Sb-doped SnO_2_ active layer forms a porous nanostructure on the Ti substrate, providing a large electrolyte-contact area and short ion-transport pathways. The lattice spacing of 0.33 nm observed in the HRTEM image corresponds to the (110) plane of SnO_2_. EDS and elemental mapping reveal Sn, Sb, O, and Ti elements; Sn, Sb, and O confirm the formation of the Sb-SnO_2_ active layer, while the Ti signal mainly originates from the Ti sheet support and the interfacial region.

**Figure 13:**
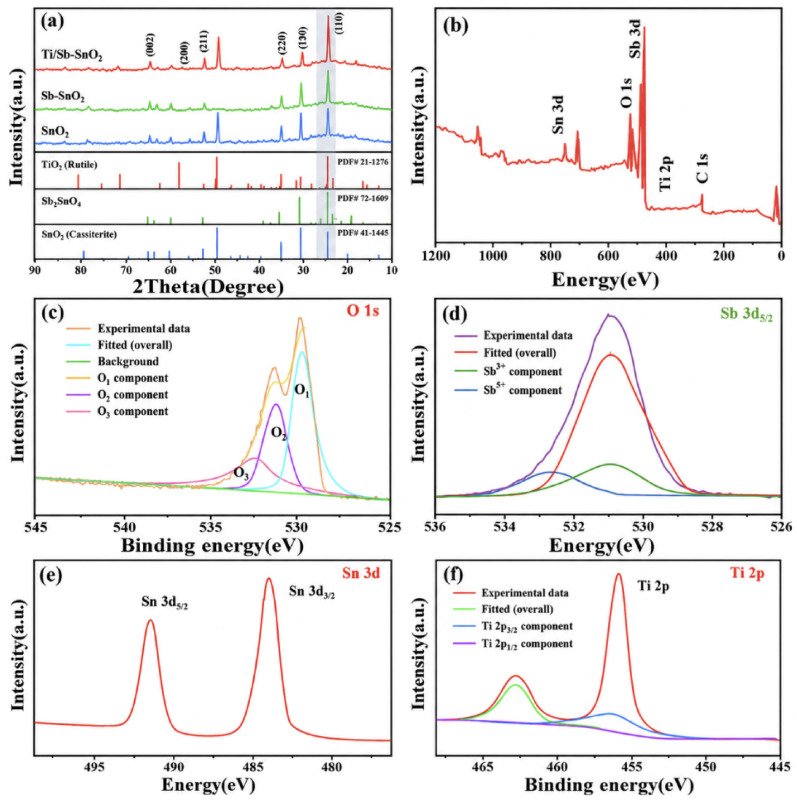
(**a**) XRD patterns, (**b**) XPS survey spectrum, and (**c**–**f**) high-resolution O 1s, Sb 3d, Sn 3d, and Ti 2p XPS spectra of SnO_2_, Sb-SnO_2_, and Ti/Sb-SnO_2_.

XRD and XPS results further reveal the phase structure and surface chemistry of Ti/Sb-SnO_2_ (Figure 13). SnO_2_, Sb-SnO_2_, and Ti/Sb-SnO_2_ all show SnO_2_-related diffraction peaks, and the experimental patterns are compared with standard reference cards for SnO_2_ (cassiterite, PDF# 41-1445), Sb_2_SnO_4_ (PDF# 72-1690), and rutile TiO_2_ (PDF# 21-1276), indicating that Sb doping does not destroy the host SnO_2_ phase. The XPS survey spectrum contains Sb 3d, Sn 3d, O 1s, Ti 2p, and C 1s signals. O 1s can be divided into lattice oxygen, defect/hydroxyl oxygen, and adsorbed oxygen components. Sn 3d_5_/_2_ and Sn 3d_3_/_2_ peaks at approximately 486.4 and 494.8 eV correspond to Sn^4+^. Sb-related peaks indicate that Sb participates in Sb-O/Sn-O bonding in an oxidized state and may induce defect formation. The Ti 2p signal is mainly associated with the Ti substrate or the interfacial oxide layer; without depth-profile XPS, ICP analysis, or lattice-parameter evidence that excludes substrate effects, it should not be used as direct evidence for Ti incorporation into the SnO_2_ lattice.

**Figure 14:**
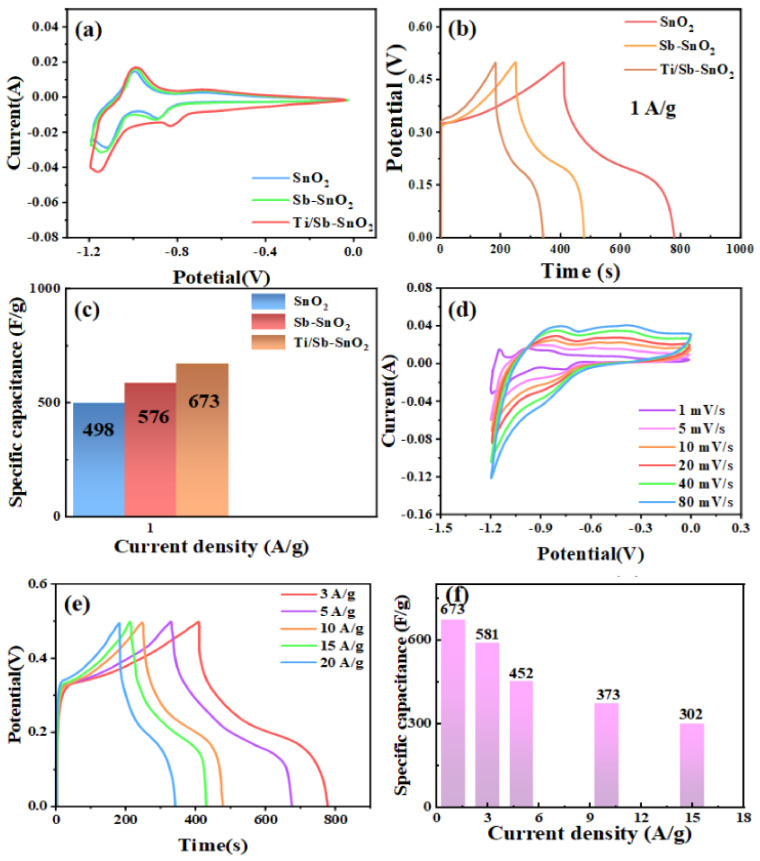
Electrochemical performance of SnO_2_, Sb-SnO_2_, and Ti/Sb-SnO_2_ negative electrode materials: (**a**) CV curves of SnO_2_, Sb-SnO_2_, and Ti/Sb-SnO_2_; (**b**) GCD curves at 1 A/g; (**c**) specific capacitances at 1 A/g; (**d**) CV curves of Ti/Sb-SnO_2_ at different scan rates; (**e**) GCD curves of Ti/Sb-SnO_2_ at different current densities; and (**f**) rate performance of Ti/Sb-SnO_2_.

**Figure 15:**
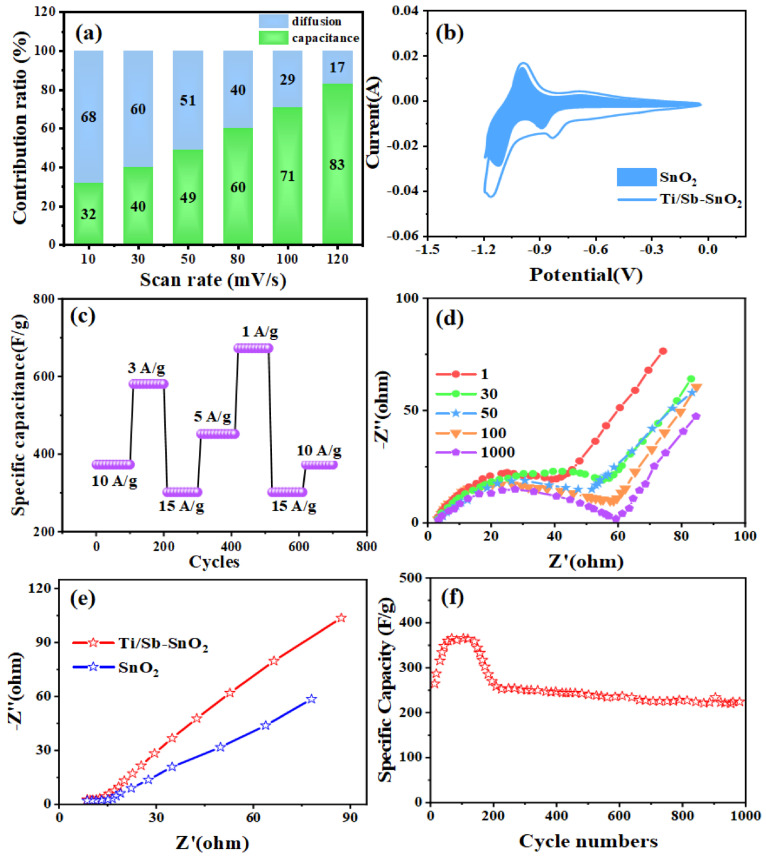
Electrochemical performance of the Ti/Sb-SnO_2_ negative electrode: (**a**) charge-storage contribution ratios at different scan rates; (**b**) CV comparison of SnO_2_ and Ti/Sb-SnO_2_; (**c**) rate performance at different current densities; (**d**) EIS spectra after different cycles; (**e**) EIS comparison of SnO_2_ and Ti/Sb-SnO_2_; and (**f**) cycling stability of the Ti/Sb-SnO_2_ electrode.

The electrochemical performance of the negative electrode is presented in Figure 14 and Figure 15. Compared with SnO_2_ and Sb-SnO_2_, Ti/Sb-SnO_2_ exhibits a larger CV-enclosed area and longer GCD discharge time, demonstrating that Sb doping, the Ti-supported current-collector configuration, and improved interfacial contact enhance the charge-storage capability of SnO_2_. Ti/Sb-SnO_2_ delivers a specific capacitance of 673 F g^−1^ at 1 A g^−1^, higher than SnO_2_ (498 F g^−1^) and Sb-SnO_2_ (576 F g^−1^), and retains 302 F g^−1^ when the current density increases to 15 A g^−1^, indicating good rate capability.

Kinetic analysis shows that when the scan rate increases from 10 to 120 mV s^−1^, the capacitive contribution of Ti/Sb-SnO_2_ increases from 32% to 83%, revealing that fast surface reactions dominate at high scan rates. During rate cycling, the electrode maintains approximately 373 F g^−1^ during the first 100 cycles at 10 A g^−1^ and recovers to approximately 365 F g^−1^ at 10 A g^−1^ after 700 rate cycles. EIS results demonstrate that Sb doping, defect regulation, and the Ti-supported current-collector/interfacial-contact configuration jointly reduce charge-transfer resistance and optimize ion-diffusion pathways, thereby improving the rate capability and structural stability of the negative electrode.

### 3.4. Performance of the La-ZnCo_2_O_4_/MnCo-LDH//Ti/Sb-SnO_2_ Asymmetric Supercapacitor

**Figure 16:**
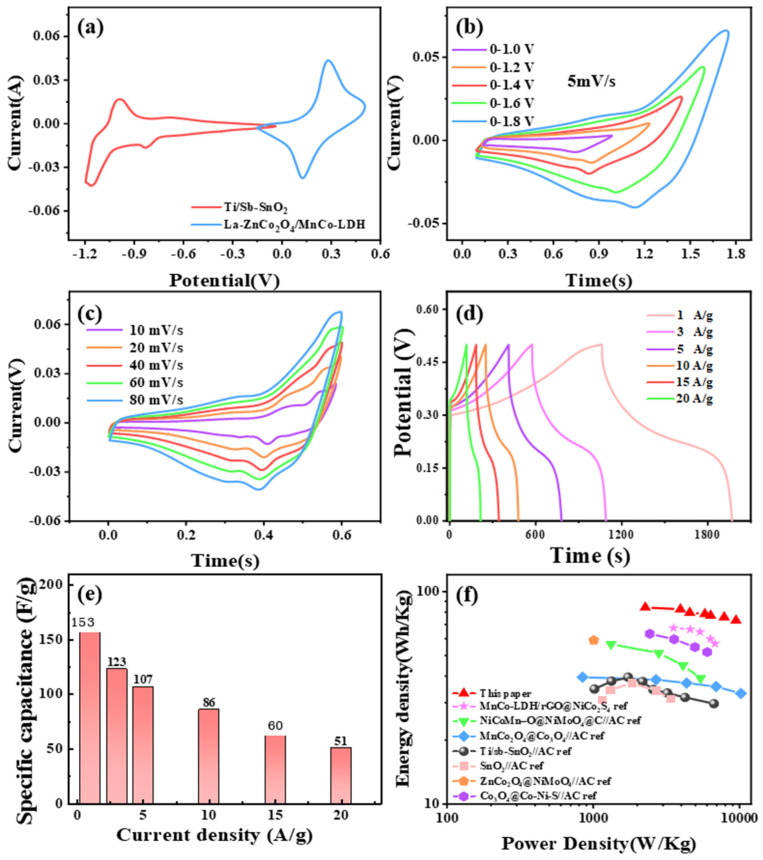
Electrochemical performance of the La-ZnCo_2_O_4_/MnCo-LDH//Ti/Sb-SnO_2_ asymmetric supercapacitor: (**a**) CV curves of the positive and negative electrodes; (**b**) CV curves of the device at different voltage windows; (**c**) CV curves of the device at different scan rates; (**d**) GCD curves at different current densities; (**e**) specific capacitances at different current densities; and (**f**) Ragone plot of the device.

Based on positive/negative electrode charge matching, an asymmetric supercapacitor was assembled using La-ZnCo_2_O_4_/MnCo-LDH as the positive electrode and Ti/Sb-SnO_2_ as the negative electrode. Figure 16a shows that the working potential windows of the two electrodes in 2 M KOH are complementary, which is favorable for widening the device voltage window and improving energy density. Voltage-window tests demonstrate that the device maintains stable CV responses within 0–1.8 V without obvious polarization, confirming the feasibility of this operating voltage.

The CV curves of the device at different scan rates show synchronous increases in peak current with increasing scan rate, indicating rapid charge response. The nearly symmetric GCD profiles indicate good charge–discharge reversibility. Based on the total active mass of both electrodes, the device delivers approximately 153 F g^−1^ at 1 A g^−1^, retains approximately 60 F g^−1^ at 15 A g^−1^, and maintains approximately 51 F g^−1^ at 20 A g^−1^, demonstrating strong rate capability. According to E = C_device(ΔV)^2^/7.2 and P = 3600E/Δt, the device achieves a maximum energy density of approximately 69 Wh kg^−1^; at 15 A g^−1^, it still maintains approximately 27 Wh kg^−1^ with a corresponding power density of approximately 13,500 W kg^−1^, showing a favorable energy–power output balance compared with recently reported related asymmetric supercapacitor systems [10,43,44,45,46,47,48].

## 4. Conclusions

La-ZnCo_2_O_4_/MnCo-LDH nanoflowers were prepared through La introduction/doping regulation, heterocomponent coupling, and ethylenediamine-assisted morphology regulation, while Ti-supported Sb-doped SnO_2_ was constructed as the negative electrode to assemble a La-ZnCo_2_O_4_/MnCo-LDH//Ti/Sb-SnO_2_ asymmetric supercapacitor. La introduction and ethylenediamine regulation transformed ZnCo_2_O_4_/MnCo-LDH from a stacked nanosheet structure into a porous nanoflower architecture, increasing the specific surface area to 382.5 m^2^ g^−1^ and improving electronic structure and charge-transfer kinetics through defect-related oxygen species. In the Ti/Sb-SnO_2_ negative electrode, Sb doping, defect regulation, and the Ti-supported current-collector/interfacial-contact configuration jointly enhanced electron transport, ion diffusion, and structural stability. Electrochemical measurements showed that the La-ZnCo_2_O_4_/MnCo-LDH positive electrode delivered 2130 F g^−1^ at 1 A g^−1^ and retained 1993 F g^−1^ after 10,000 cycles at 3 A g^−1^. The Ti/Sb-SnO_2_ negative electrode delivered 673 F g^−1^ at 1 A g^−1^ and retained 302 F g^−1^ at 15 A g^−1^. The assembled device operated stably from 0 to 1.8 V, achieved a maximum energy density of approximately 69 Wh kg^−1^, and maintained approximately 27 Wh kg^−1^ at 15 A g^−1^ with a power output of approximately 13,500 W kg^−1^. These results demonstrate that the synergistic engineering of rare-earth introduction, defect regulation, three-dimensional porous architecture, and positive/negative electrode charge matching is an effective route for improving transition-metal oxide/LDH-based asymmetric supercapacitors.

## Data Availability

The data presented in this study are available on request from the corresponding author.

## Abbreviations

The following abbreviations are used in this manuscript:

ASC	Asymmetric supercapacitor
CV	Cyclic voltammetry
EDS	Energy-dispersive X-ray spectroscopy
EIS	Electrochemical impedance spectroscopy
GCD	Galvanostatic charge–discharge
HRTEM	High-resolution transmission electron microscopy
LDH	Layered double hydroxide
SEM	Scanning electron microscopy
TEM	Transmission electron microscopy
XPS	X-ray photoelectron spectroscopy
XRD	X-ray diffraction
